# Postoperative physical rehabilitation in the elderly patient after emergency surgery. Influence on functional, cognitive and quality of live recovery: study protocol for a randomized clinical trial

**DOI:** 10.1186/s13063-024-08406-0

**Published:** 2024-09-04

**Authors:** Irene Esquiroz Lizaur, Fabricio Zambom-Ferraresi, Fabiola Zambom-Ferraresi, Iranzu Ollo-Martínez, Antón De la Casa-Marín, Nicolás Martínez-Velilla, Ana Recreo Baquedano, Arkaitz Galbete Jimenez, Gregorio González Alvarez, María Concepción Yarnoz Irazabal, Inés Eguaras Córdoba

**Affiliations:** 1 Surgery Department, Hospital Universitario de Navarra (HUN), Pamplona, Navarra Spain; 2 Department of Health Sciences, Public University of Navarra (UPNA), Pamplona, Navarra Spain; 3 Biomedical Research Centre of the Government of Navarre (Navarrabiomed), Navarra Institute for Health Research (IdiSNA), Pamplona, Navarra Spain; 4 CIBER of Frailty and Healthy Aging (CIBERFES), Instituto de Salud Carlos III, Madrid, Spain; 5 Geriatric Department, Hospital Universitario de Navarra (HUN), Pamplona, Navarra Spain

**Keywords:** Rehabilitation, Urgent general surgery, Geriatric intervention, Recovery of function, Randomized controlled trial

## Abstract

**Background:**

The progressive aging of the population has meant the increase in elderly patients requiring an urgent surgery. Older adults, especially those with frailty, have a higher risk for complications, functional and cognitive decline after urgent surgery. These patients have their functional and physiological reserve reduced which makes them more vulnerable to the effects of being bedridden. The consequences are at multiple levels emphasizing the functional loss or cognitive impairment, longer stays, mortality and institutionalization, delirium, poor quality of life and increased use of resources related to health.

We aim to determine whether postoperative physical rehabilitation can prevent functional and cognitive decline and modify the posterior trajectory.

**Methods/design:**

This study is a randomized clinical trial, simple blinded, conducted in the Department of Surgery of a tertiary public hospital in Navarra (Hospital Universitario de Navarra), Spain. Patients >  = 70 years old undergoing urgent abdominal surgery who meet inclusion criteria will be randomly assigned to the intervention or control group. The intervention will consist of a multicomponent physical training programme, which will include progressive and supervised endurance, resistance and balance training for 4 weeks, twice weekly sessions with a total of 8 sessions, and the group control will receive the usual care. The primary outcome measure is the change in functional (SPPB) and cognitive status (Mini-Mental State Examination) and the change of quality of life (EuroQol-5D-VAS) during the study period. The secondary outcomes are postoperative complications, length of stay, delirium, mortality, use of health resources, functional status (Barthel Index and handgrip strength tests), cost per quality-adjusted life year and mininutritional assessment. The data for both the intervention group and the control group will be obtained at four different times: the initial visit during hospital admission and at months 1, 3 and 6 months after hospital discharge.

**Discussion:**

If our hypothesis is correct, this project could show that individualized and progressive exercise programme provides effective therapy for improving the functional capacity and achieve a better functional, cognitive and quality of life recovery. This measure, without entailing a significant expense for the administration, probably has an important repercussion both in the short- and long-term recovery, improving care and functional parameters and could determine a lower subsequent need for health resources. To verify this, we will carry out a cost-effectiveness study.

The clinical impact of this trial can be significant if we help to modify the traditional management of the elderly patients from an illness model to a more person-centred and functionally oriented perspective. Moreover, the prescription of individualized exercise can be routinely included in the clinical practice of these patients.

**Trial registration:**

ClinicalTrials.gov Identifier: NCT05290532. Version 1. Registered on March 13, 2022.

**Supplementary Information:**

The online version contains supplementary material available at 10.1186/s13063-024-08406-0.

## Background

The population in developed countries is rapidly aging; the number of Americans aged 65 years or older was 43 million in 2012, and this number is expected to be more than doubled by 2060 [1]. The United Nations in its report “World Population Prospects 2019: Highlights” estimates that, in 2050, one in six people in the world will be over 65 years old (16%) [2]. Specifically in Spain, in 2050, people over 65 will represent more than 30% of the total population and octogenarians will exceed 4 million [3]. This rapid rate of population aging has been outpaced by an increase in number of older patients needing surgical intervention as a main modality of treatment [4–6].

There still exists lack of consensus about the definition of what age is considered the cutoff for geriatrics (65 vs 70 vs 75 years old), and there is consensus that patients should not be treated based on their age alone [7]. However, there is evidence that age-related psychophysiological changes and co-morbidities affect older people’s tolerance to surgery, becoming a major life event with the risk of permanent and definitive disabilities [8]. Frailty is a clinical syndrome defined by vulnerability and an increased risk of the individual to develop negative health-related events such as disability and/or mortality under external stressor factors such as surgery [9]. To identify older adults at high risk of numerous adverse outcomes, five criteria have been established weight loss, exhaustion, leisure-time activity, gait speed and grip strength [10].

Related to this, quality of life has become one of the main health goals of the twenty-first century. Older adults consistently indicate that maintaining independent function is their top priority, more than 70% of the elderly would not choose a treatment that would lead to severe functional impairment, even if we ensured their survival [11–14]. Hospital admission in these patients often leads to significant functional impairment; between 20 and 46% of patients have functional loss in one or more activities of daily life [11, 15–17]. Of these patients who present functional deterioration at discharge, recovery of the baseline situation is achieved in only 30% of them, most within the first month [18].

To reduce functional decline in older adults undergoing elective surgery, multimodal rehabilitation programmes ERAS (Enhanced Recovery After Surgery) have been designed. Several previous studies have included supervised exercises as multimodal rehabilitation; these programmes aim to promote postoperative recovery in elective surgery. They have subsequently been extrapolated to urgent surgery proving that once adapted they are effective [19–21].

Focusing on the elderly patient undergoing surgery, the functional recovery is severely affected in the subgroup of patients who have required urgent surgery and in those with post-surgical complication [16, 17, 22]. In urgent surgery patients, prehabilitation programme cannot be performed, although post-surgical rehabilitation is feasible. Rehabilitation based on physical exercise during admission in elderly patients after an acute process has shown an earlier recovery of their baseline functional status [23–28]. However, there are few recommendations for rehabilitation programmes including physical activity in older patients after urgent surgery.

We developed the first randomized clinical trial to assess the effectiveness of a rehabilitation programme based on physical exercise during the first month after surgery.

The main objective of this study is to analyze whether postoperative physical rehabilitation in a short period of time improves functional and cognitive recovery and long-term quality of life (6 months) in elderly adults undergoing urgent abdominal surgery.

## Methods/design

### Study design

This study is a parallel group, two-arm, superiority randomized trial with 1:1 allocation ratio, is monocentric and is conducted in the Department of Surgery of a tertiary public hospital in Navarra (Hospital Universitario de Navarra), Spain. Patients undergoing urgent abdominal surgery who meet inclusion criteria will be randomly assigned to the intervention or control group.

Patient recruitment will begin in 4 days after the surgical procedure, and these patients will be identified through the list of patients admitted to the hospital and assigned to the Department of General Surgery. Prior to randomization, the investigators will review the contraindications to participate in the exercise programme and will provide general information about the study. After signing an informed consent form, the subjects will be chosen randomly. 

Randomization will be performed by applying http://www.randomizer.org/. The doctor who decides the inclusion in the intervention or control group will not be the attending physician. Patients will be informed of the random inclusion in one of the groups.

The information in both groups is obtained in four different stages: the initial visit and at months 1, 3 and 6 after hospital discharge.

The protocol employs relevant standard protocol items for clinical trials according to the SPIRIT 2013 statement [29] and follows the CONSORT statement [30] for transparent reporting.

The trial is registered at ClinicalTrials.gov, identifier NCT05290532.

### Study participants and eligibility criteria

Individuals ≥ 70 years old admitted to the Department of General Surgery of the Hospital Universitario de Navarra after an urgent surgery between March 2022 and March 2025.

**Fig. 1:**
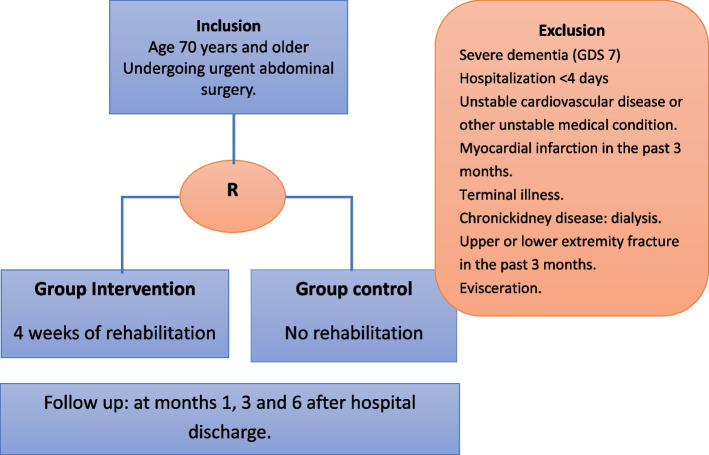
Inclusion and exclusión criteria, groups and follow-up

The inclusion criteria are (Fig. 1):

- Age 70 years and older
- Undergoing urgent abdominal surgery
- Able to ambulate, with or without personal / technical assistance or move unassisted in a wheelchair
- Able to communicate: English or Spanish
- Barthel Index > 60.
- Informed consent: must be capable and willing to provide consent.

The exclusion criteria are (Fig. 1):

- Severe dementia (GDS 7)
- Unwillingness to either complete the study requirements or to be randomized into control or intervention group
- Unstable cardiovascular disease or other unstable medical condition
- Myocardial infarction in the past 3 months
- Terminal illness
- Chronic kidney disease: dialysis
- Upper or lower extremity fracture in the past 3 months
- Evisceration
- Patients transferred to a rehabilitation clinic prior to home discharge

If the patient throughout the study presents any of the exclusion criteria and wishes to leave the study, they will be removed from the study.

### Randomization and blinding

The study participants will be randomized into an intervention group and a control group following a simple randomization procedure with a 1:1 allocation through a computer system (www.randomizer.org). The doctor who decides the inclusion in the intervention or control group will not be the attending physician, and the assessment staff will be blinded to the participant randomization assignment. It will not be possible to conceal the group assignment from the staff involved in the training of the intervention group. Due to the nature of the study, patients may not be blinded as to the group to which they belong.

### Sample size and statistical analysis

Assuming a type I error of 0.05, a correlation between pre and post-intervention values of the Short Physical Performance Battery (SPPB) of *r* = 0.6 and a standard deviation for the SPPB of 2.5, the required sample size to detect with a power of 90% a minimum difference of 1 point between groups in the change of SPPB score is 87 patients per group [27]. Assuming losses of 20%, the final objective of the size of each group is 109 patients, and, consequently, a total sample size will be 218 subjects. For the estimation, an ANCOVA method for the analysis of the differences has been considered. The outcome variables will be analyzed using mixed models, without missing data imputation. If the proportions of missing data are very large (more than 40%) on important variables, then trial results will be considered as hypothesis-generating results.

Baseline values will be compared by group using descriptive statistics as mean and standard deviation or median and interquartile range for quantitative variables and frequencies and percentages for categorical ones. To determine the efficacy of the intervention in the quantitative variables, such as the SPPB, we will use ANCOVA models, using post-intervention value as dependent variable, group study as the principal effect and pre-intervention value as covariate. If relevant group differences were observed at baseline, we would adjust for these variables in the model. In the case of qualitative or categorized variables (such as whether an improvement of a given magnitude between pre and post-intervention has been achieved or not), comparisons between groups will be conducted with the chi-square test or Fisher’s test, and complemented with logistic regression if additional adjustment is needed.

The level of statistical significance will be 0.05. Data will be analyzed using an intention-to-treat approach and using SPSS and R statistical software.

### Methods for further analysis

Subgroup analyses will be performed to understand whether exercise is more or less effective according to age, type of surgery, comorbidity or hospital stay. These subgroup analyses will follow the same plan as the primary analysis, as well as their interaction with the experimental condition.

### Plans for communicating important protocol amendments to relevant parties

Ethical approval to conduct this study has been granted by the Hospital Universitario de Navarra Research Board (PI_2021/39). If relevant, current participants will be informed of protocol modifications. The ClinicalTrials.gov registry for this study will be updated with important protocol amendments.

### Data collection and management

Completed personal data or other documents containing protected personal health information will be kept in a locked file at the principal investigator office in the Hospital Universitario de Navarra. Data will be entered into an electronic de-identified database by authorized study team members, and checked for completeness and accuracy. Access to data with identifiers will be restricted to authorized study team members and authorities. Any data required to support the protocol can be supplied on request. Identifiable data will be destroyed 10 years after study finalization or 5 years after the last publication.

The datasets analyzed during the current study and statistical code are available from the corresponding author on reasonable request, as is the full protocol.

Adherence to training in the intervention group will be monitored by exercise trainers who will track attendance in training sessions. If participants miss any training sessions, they will be offered make-up sessions to complete the full 8 sessions of training. In both groups, in order to ensure attendance at the consultation, they are informed of the appointment by telephone and by letter.

### Detail description

Participants will be randomly assigned to the following groups:

- Usual care group (control):

Participants randomly assigned to the usual care group will receive normal hospital care based on the Enhanced Recovery After Surgery (ERAS) protocol [31], including physical rehabilitation when needed.

- Multicomponent exercise group (intervention):

The intervention will consist of a multicomponent physical training programme, which will include progressive and supervised endurance, resistance and balance training for 4 weeks, twice weekly sessions with a total of 8 sessions.

The supervised multicomponent exercise training programme will be comprised of 5 min of endurance training on a cycle ergometer, followed by upper and lower body resistance exercises, tailored to the functional capacity of the individual, using weight machines and with the goal of 2–3 sets of 8–10 repetitions at an intensity of 40–60% of 1 maximal repetition (1RM) (Matrix, Johnson Health Tech, Ibérica, SL, Madrid, Spain) combined with balance exercises and stretching. Each resistance training session will include chair squat and hip abduction exercises, as well as training on variable resistance machines with two exercises for the lower extremities (leg press and knee extension) and two exercises for the upper extremities (seated chest press and seated row). The training protocol is shown in Table 1.

**Table 1:**
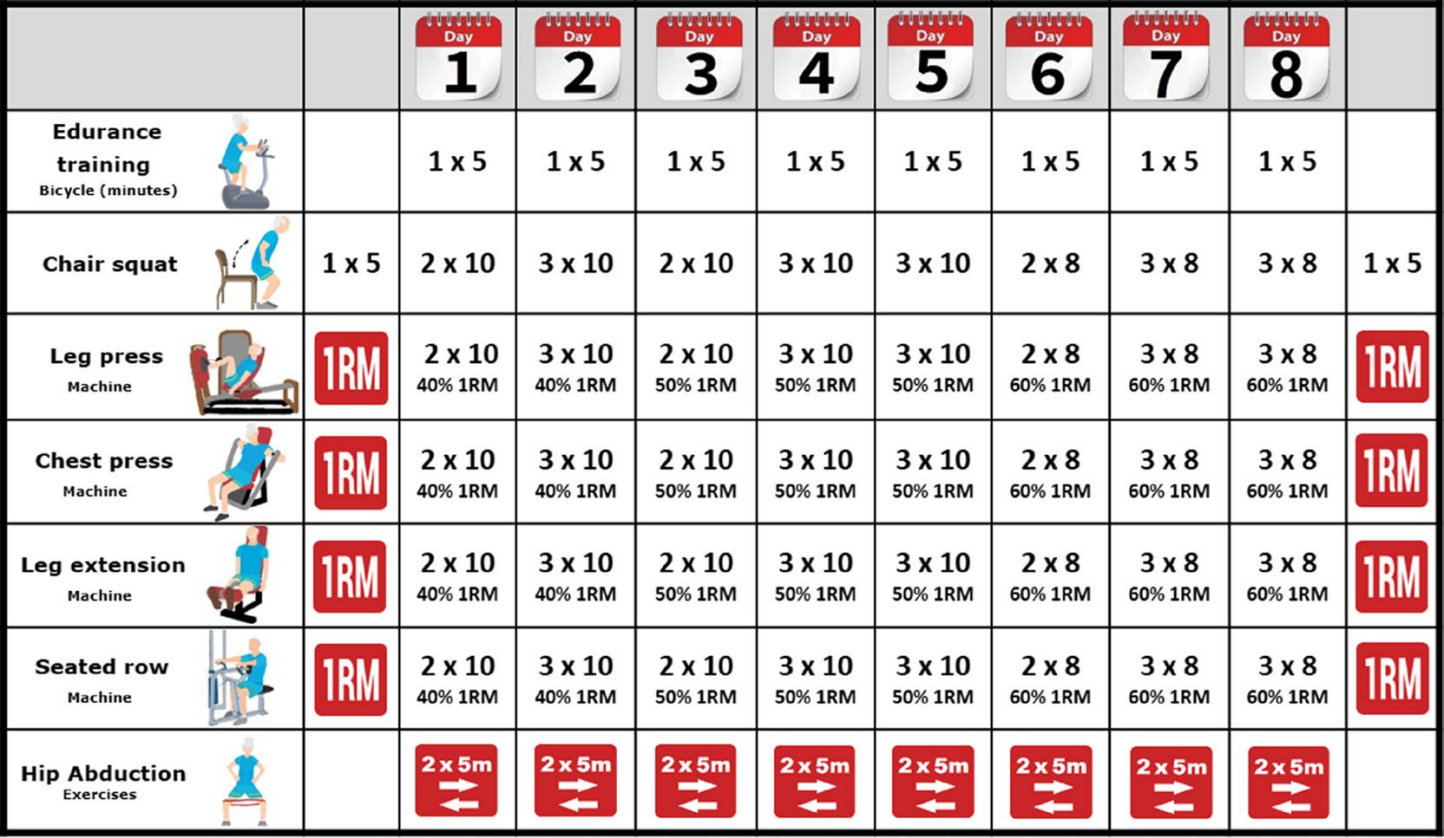
Intervention group exercise

One repetition maximum

Forward (to the right) and back (to the left) twice

Study staff will collect all adverse events that will be noted, which includes any event that occurs during or up to 15 min, after intervention, and persists despite therapy interruption and constitute criteria for discontinuing intervention. Severe adverse events will be promptly reported to the Regional Ethics Committee of the HUN. Management of adverse effects will be based on participant protection and safety.

If participants desire to stop training or develop health conditions or injury that precludes safe participation of exercise over the course of the intervention, they will be excluded from the study. 

Participants in either the intervention or control group will be asked to not participate in another structured exercise regimen or intervention over the course of their participation; otherwise, they will not be allowed to continue participation.

### Outcome measures

#### Primary outcome

The primary outcome measure is the change in functional and cognitive status and the change of quality of life during the study period. The functional capacity of patients will be evaluated by the Short Physical Performance Battery (SPPB) (Annex 1), which combines balance, gait ability and leg strength using a single tool. The total score will range from 0 (worst) to 12 points (best). The SPPB test has been shown to be a valid instrument for screening frailty and predicting disability, institutionalization and mortality. The magnitude of meaningful change was a one-point change in the score which has clinical relevance. If the total score is less than 10, it indicates frailty and a high risk of disability and falls.

The capacity will be assessed with the Mini-Mental State Examination (MMSE) (Annex 2). This examination is composed of seven categories designed to assess specific cognitive functions: orientation to time (5 points), orientation to place (5 points), registration of three words (3 points), attention and calculation (5 points), recalling the three words (3 points), language (8 points) and constructive visual capacity (1 point).

The MMSE score ranges from zero to 30 points, and lower values indicate possible cognitive deficit. Values from 27 to 30 denote preserved cognitive functions; from 24 to 26, changes that do not suggest deficit; from 20 to 23, changes that suggest cognitive deficit. Scores from 20 to 26 represent mild cognition impairments; between 11 and 20, moderate cognition impairment; and scores under 10 represent severe cognition impairments.

Changes in quality of life will be assessed by EuroQol-5D-VAS (Annex 3). This is a generic health status questionnaire, consisting of five dimensions (mobility, self-care, usual activities, pain/ discomfort, anxiety/depression) including three responses. It also includes a visual analogue scale for recording an individual’s rating of their current health-related quality of life (scale 0 to 100).

#### Secondary outcome measure

- Postoperative complications: Clavien Dindo (Annex 4) and Comprehensive Complication Index (Annex 5)
- Length of stay
- Delirium: Confusion Assessment Method (CAM) (Annex 6)
- Mortality: number of days alive after admission to the hospital
- Use of health resources: new admission to the hospital, admission to nursing homes and visits to the general practitioner
- Functional status: Barthel index (Annex 7)
- Cost per quality-adjusted life year: both direct and indirect study participant costs
- Mininutritional Assessment short form (MNA) (Annex 8)
- Handgrip strength test

Surgeons and physiotherapists will help participants to fill in the questionnaires. The information in both groups is obtained in a face-to-face consultation in four different stages: the initial visit and at months 1, 3 and 6 after hospital discharge. The questionnaires and tests are easy to perform, and the studio staff is qualified to perform them in a homogeneous way. The scales that we used to evaluate functional capacity [32, 33], mental state [34], quality of life [35] and nutritional status [36] have been validated for use in Spain.

**Table:** 

Assessments	During hospital admission	1 month	3 months	6 months
**SPPB**	X	X	X	X
**Handgrip strength test**	X	X	X	X
**MMSE score**	X	X	X	X
**EuroQol-5D-VAS**	X	X	X	X
**Barthel Index**	X	X	X	X
**Mininutritional assessment short form**	X	X	X	X
**Confusion assessment method**	X			
**Clavien Dindo**	X	X		
**Comprehensive Complication Index**	X	X		
**Length of stay**	X			
**Use of health resources**		X	X	X
**Mortality**	X	X	X	X

### Participant timeline

**Fig. 2:**
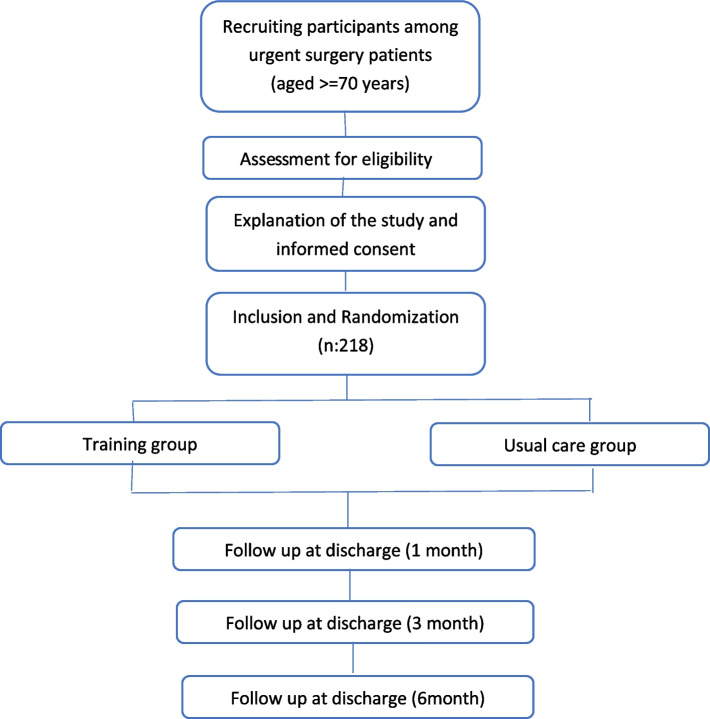
Flow diagram of the study protocol

**Fig. 3:**
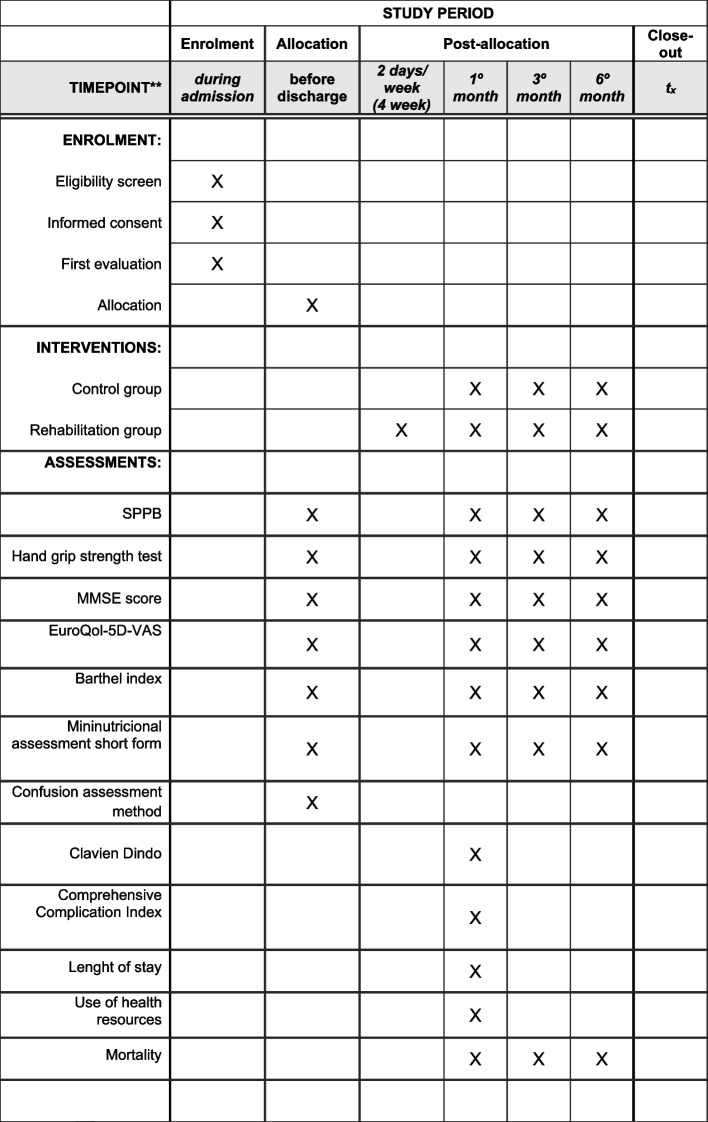
SPIRITE figure enrolment, interventions, and assessments

The schedule of registration, interventions, evaluations and visits for the participants is shown in the following diagrams (Figs. 2 and 3).

## Opportunity of the trial/discussion

Functional decline and impaired quality of life are the main adverse outcomes of urgent surgery in the elderly patients. A high percentage of patients lose their autonomy, increasing the need for care at home and even the patient’s institutionalization in a postoperative rehabilitation clinic. All this translates into a psychological impact for the patient and his or her environment and in a considerable unquantified increase in health spending.

All the current evidence from international organizations’ works reminds us that the most important thing in the elderly is to focus attention and health care on the maintenance of intrinsic capacity, that is, its functional capacity.

However, surgeons are focused on medical-surgical problems during hospitalization, being less attentive to functional recovery, which would require longer hospital stays and would greatly increase healthcare costs.

The rehabilitation programme that we are proposing in this study could be applied in daily clinical practice, incorporating elderly patients undergoing urgent abdominal surgery into protocolized and standardized rehabilitation programmes with postoperative physical activity.

An important aspect of our trial is the inclusion of elderly patients after an urgent abdominal surgery; the majority of trials with aged frail participants excluded the patients with a recent surgery; however, the surgery is a major factor in the loss of functionality. This trial could demonstrate that a supervised multicomponent exercise training programme adapted to each patient can be performed safely after emergency surgery and could improve functional results.

If our hypothesis is correct, this project could show that individualized and progressive exercise programme provides effective therapy for improving the functional capacity and achieve a better functional, cognitive and quality of life recovery. This measure, without entailing a significant expense for the administration, probably has an important repercussion both in the short and long term, improving care and functional parameters and could determine a lower subsequent need for health resources. To verify this, we will carry out a cost-effectiveness study.

The clinical impact of this trial can be significant if we help to modify and shift the traditional management of this population from an illness model to a more person-centred and functionally oriented perspective. In this way, the prescription of individualized exercise could be routinely included in the clinical practice of these patients.

## Trials status

This is the first and definitive protocol version. The trial commenced recruitment in March 2022 and is currently open for recruitment. Recruitment will cease when 218 participants have been randomized.

## Dissemination

The results of our study will be disseminated via presentations at international conferences and articles in peer-reviewed journals. The study will be implemented and reported in accordance with the Standard Protocol Items: Recommendations for Interventional Trials (SPIRIT) guidelines.

## Supplementary Information

Additional file 1. SPIRIT checklist for trials.

Additional file 2. Annexes 1 to 9.

## Data Availability

All members of the Study group will have access to the anonymised, cleaned data set upon completion of the final post-intervention testing after approval from the steering committee.

## Abbreviations

1RM: One maximal repetition
CAM: Confusion assessment method
ERAS: Enhanced Recovery After Surgery
GDS: Global Deterioration Scale
MMSE score: Mini-Mental State Examination
MNA-SF: Mininutritional assessment short form
SPPB: Short Physical Performance Battery
