# Supplementary material for: Postoperative physical rehabilitation in the elderly patient after emergency surgery. Influence on functional, cognitive and quality of live recovery: study protocol for a randomized clinical trial
Source: Trials. 2024 Sep 4;25:584. doi: 10.1186/s13063-024-08406-0 (PMC11373425; doi:10.1186/s13063-024-08406-0)
Supplement: Supplementary file 2 — Additional file 2. Annexes 1 to 9. [file 13063_2024_8406_MOESM2_ESM.docx]

**Annex1. Short Physical Performance Battery (SPPB)**


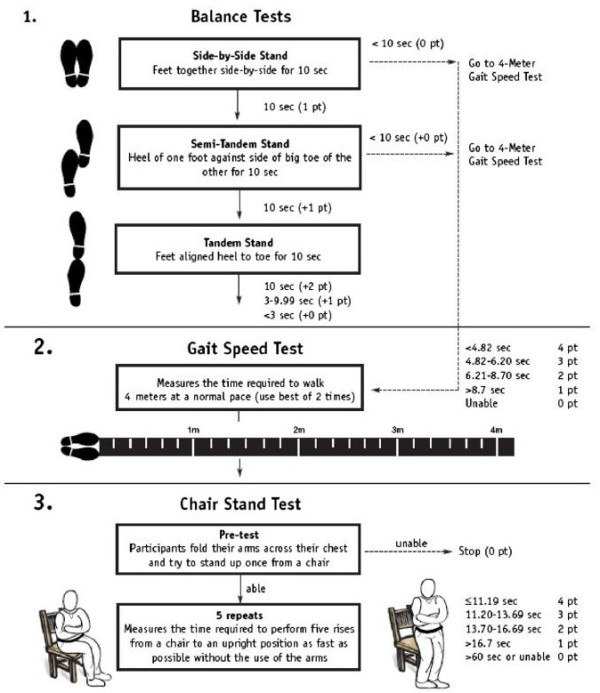


- **Short performance physical battery test (SPPB) based on Riskowski JL et al**

**Annex 2: Mini Mental State Examination (MMSE)**


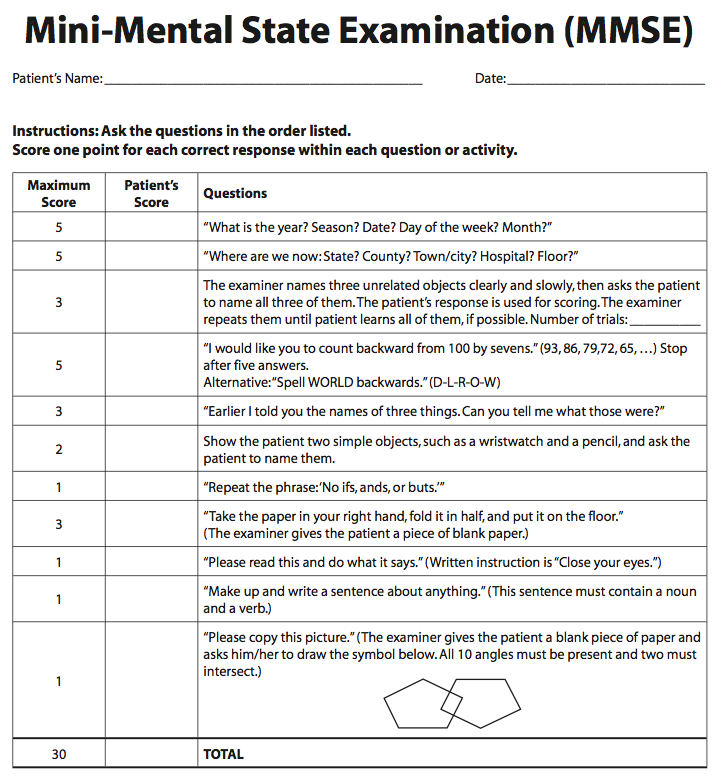


**Annex 3: EuroQol-5D-VAS**


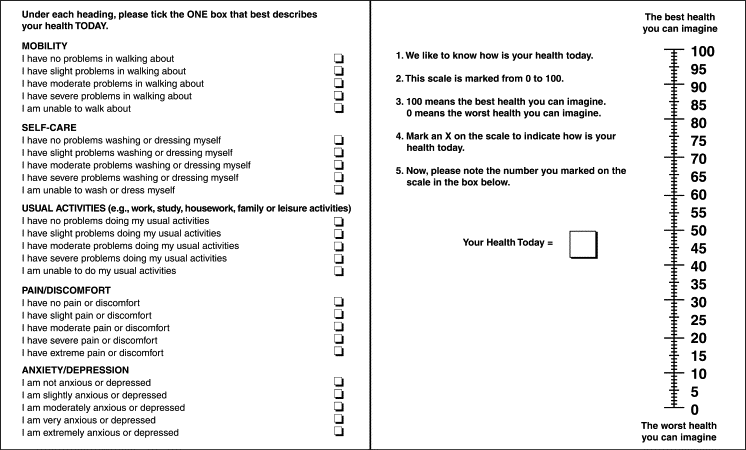


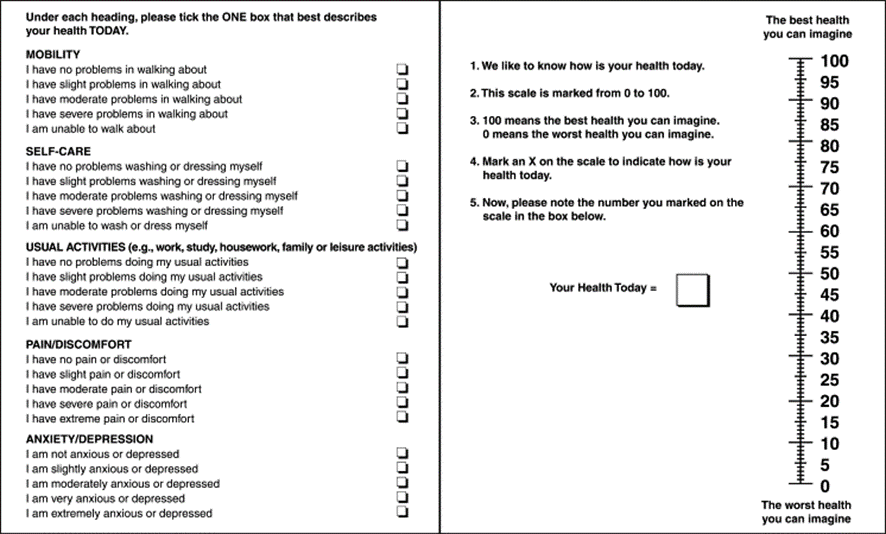


**Annex 4: Clavien-Dindo**

**
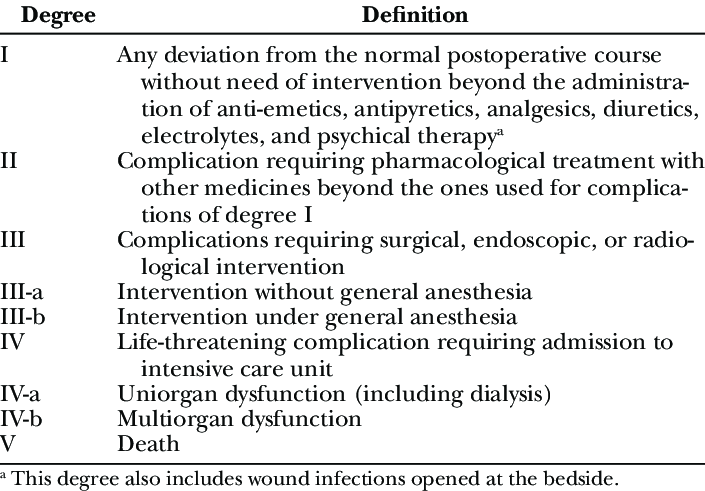
**

**Annex 5: Comprehensive Complication Index (CCI)**

**
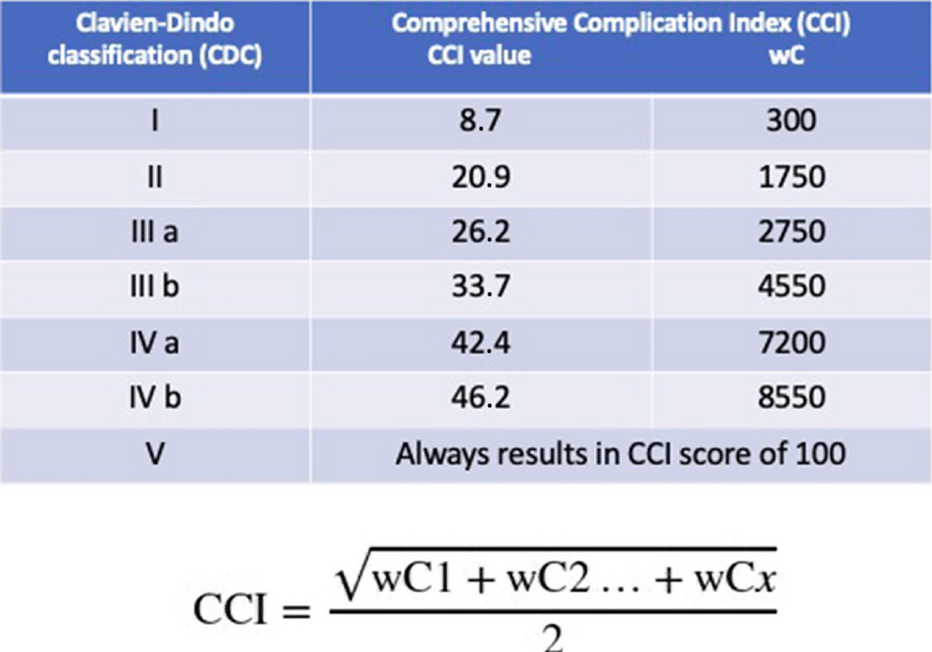
**

**Annex 6: Confusion assessment method**

**
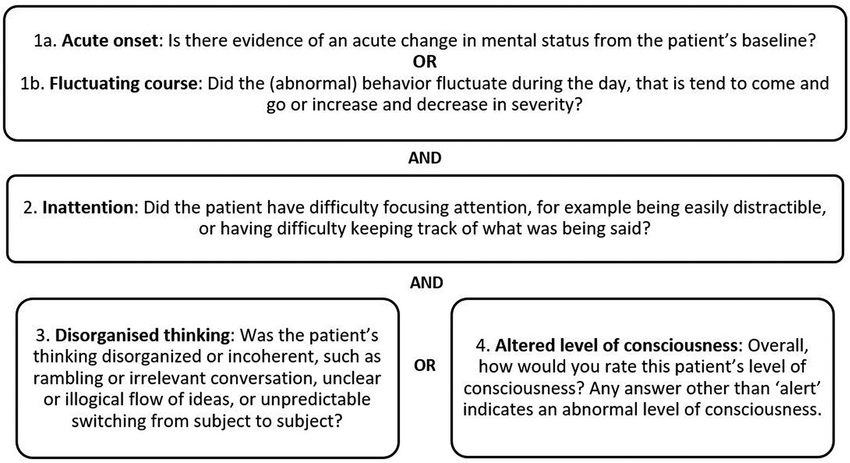
**

**Annex 7: Barthel Index**


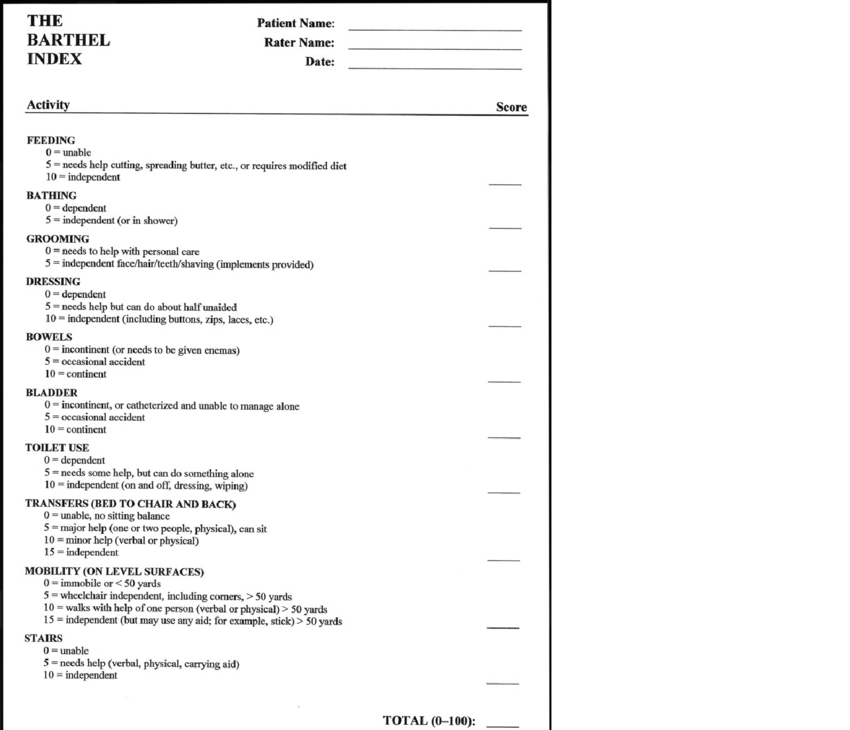


**Annex 8: Mininutricional assessment short form**

**Annex 9: Informed consent material**
